# DOLORisk: study protocol for a multi-centre observational study to understand the risk factors and determinants of neuropathic pain

**DOI:** 10.12688/wellcomeopenres.14576.2

**Published:** 2019-02-01

**Authors:** Mathilde M.V. Pascal, Andreas C. Themistocleous, Ralf Baron, Andreas Binder, Didier Bouhassira, Geert Crombez, Nanna B. Finnerup, Janne Gierthmühlen, Yelena Granovsky, Leif Groop, Harry L. Hebert, Troels S. Jensen, Kristinn Johnsen, Mark I. McCarthy, Weihua Meng, Colin N.A. Palmer, Andrew S.C. Rice, Jordi Serra, Romà Solà, David Yarnitsky, Blair H. Smith, Nadine Attal, David L.H. Bennett

**Affiliations:** 1 Nuffield Department of Clinical Neurosciences, University of Oxford, Oxford, OX3 9DU, UK; 2 Division of Neurological Pain Research and Therapy, Department of Neurology, Universitätsklinikum Schleswig-Holstein, Kiel, 24105, Germany; 3 INSERM U-987, Centre d'Evaluation et de Traitement de la Douleur, APHP, CHU Ambroise Paré, Boulogne-Billancourt, France and University Versailles Saint Quentin en Yvelines, Versailles, 78000, France; 4 Department of Experimental-Clinical and Health Psychology, Ghent University, Ghent, 9000, Belgium; 5 Department of Clinical Medicine, Danish Pain Research Center, Aarhus University, Aarhus, 8000, Denmark; 6 Department of Neurology, Aarhus University Hospital, Aarhus, 8200, Denmark; 7 Department of Neurology, Rambam Health Care Campus, Technion Faculty of Medicine, Haifa, Israel; 8 Department of Clinical Sciences, Diabetes and Endocrinology, University of Lund, Malmö, Sweden; 9 Division of Population Health Sciences, University of Dundee, Dundee, DD1 9SY, UK; 10 MentisCura, Reykjavik, 105, Iceland; 11 Wellcome Trust Centre for Human Genetics, University of Oxford, Oxford, OX3 7BN, UK; 12 Centre for Pharmacogenetics and Pharmacogenomics, Medical Research Institute, Ninewells Hospital and School of Medicine, University of Dundee, Dundee, DD1 9SY, UK; 13 Pain Research Group, Imperial College London, London, SW10 9NH, UK; 14 Neuroscience Technologies, Ltd, Barcelona, Spain

**Keywords:** pain, neuropathy, neuropathic pain, diabetes, nerve injury, risk factors, protocol

## Abstract

**Background: **Neuropathic pain is an increasingly prevalent condition and has a major impact on health and quality of life. However, the risk factors for the development and maintenance of neuropathic pain are poorly understood. Clinical, genetic and psychosocial factors all contribute to chronic pain, but their interactions have not been studied in large cohorts. The DOLORisk study aims to study these factors.

**Protocol: **Multicentre cross-sectional and longitudinal cohorts covering the main causes leading to neuropathic pain (e.g. diabetes, surgery, chemotherapy, traumatic injury), as well as rare conditions, follow a common protocol for phenotyping of the participants. This core protocol correlates answers given by the participants on a set of questionnaires with the results of their genetic analyses. A smaller number of participants undergo deeper phenotyping procedures, including neurological examination, nerve conduction studies, threshold tracking, quantitative sensory testing, conditioned pain modulation and electroencephalography.

**Ethics and dissemination: **All studies have been approved by their regional ethics committees as required by national law. Results are disseminated through the DOLORisk website, scientific meetings, open-access publications, and in partnership with patient organisations.

**Strengths and limitations:**

- Large cohorts covering many possible triggers for neuropathic pain
- Multi-disciplinary approach to study the interaction of clinical, psychosocial and genetic risk factors
- High comparability of the data across centres thanks to harmonised protocols
- One limitation is that the length of the questionnaires might reduce the response rate and quality of responses of participants

## Introduction

Neuropathic pain affects 7–10% of the general population ^1^ and has a major impact on physical health, psychological health and quality of life ^2^. The response to analgesic treatment is often inadequate with only 40–60% of patients achieving partial relief, often at the cost of adverse effects ^3^. The prevalence of neuropathic pain will increase due to the increasing prevalence of predisposing conditions, such as diabetes mellitus, and ageing, which is associated with neuropathic pain ^1^. There is an urgent clinical need to translate an increased preclinical level of understanding of neuropathic into clinical practice. In particular we need to understand the pathophysiology of neuropathic pain in clinical cohorts.

Neuropathic pain arises as a consequence of a disease or lesion in the somatosensory nervous system ^4^. However, not all patients with such a lesion develop neuropathic pain. We do not understand why only a sub-group of patients with the same disease or neurological lesion develop neuropathic pain. The severity and impact of neuropathic pain vary between individuals with similar conditions ^5^ and are unpredictable. A plausible explanation for the variation in neuropathic pain prevalence and severity is a complex interaction between genetic, psychosocial, and clinical risk factors in a vulnerable individual ^6– 8^.

A recent and significant advance in neuropathic pain research has been the development of clinical tools, such as standardised questionnaires and quantitative sensory testing for sensory phenotyping, that differentiate and stratify neuropathic pain ^9– 13^. We have entered an era whereby patients can be phenotyped in unprecedented detail in terms of sensory profile, psychological factors and physiological measures such as nerve excitability testing. We have the opportunity to combine major advances in phenotyping with genomics to improve our understanding of neuropathic pain.

## Aims and objectives

DOLORisk is a multi-centre observational study that aims to understand the risk factors and determinants for neuropathic pain.

### Primary objectives

The primary objectives of DOLORisk are (1) to identify the influence of demographic, psychological and clinical factors on the risk of developing and maintenance of neuropathic pain, and (2) to study the association of genetic factors with the risk of developing and maintaining neuropathic pain.

### Secondary objectives

DOLORisk also aims to determine if patient stratification using physiological and psychological factors can predict neuropathic pain risk and progression. Based on the analysis of these risk factors, the study will lead to the development of a risk model for neuropathic pain, combining measurable genetic and environmental factors.

## Methods

### Study design

The first step was to develop a protocol that would be used by all participating centres to identify and characterise people with neuropathic pain. The instruments chosen to phenotype DOLORisk participants were the object of a consensus meeting between the recruitment centres in October 2015. This was based on a recent international consensus on phenotyping neuropathic pain (NeuroPPIC), led by the Special Interest Group on Neuropathic Pain (NeuPSIG), of the International Association for the Study of Pain ^14^. The respective merits and reported accuracy of available scales, questionnaires and self-reported measures were discussed and the following were included in the final DOLORisk protocol ( Table 1). The DOLORisk protocol has been aligned across all recruitment centres to make data integration possible. The “core” protocol consists of questionnaires only. All participants recruited complete the core protocol and are classified according to the presence and extent of any neuropathic pain. This information will be used to look for genetic and basic clinical risk factors using the methods outlined below. The “extended” protocol consists of more detailed phenotyping and uses multiple tools. The tools used for any subject depend on the recruitment centre to which he or she is recruited ( Table 2). A sub-group of participants will be recruited through the extended protocol.

**Table 1.** Questionnaires of the DOLORisk protocol.

Category	Questionnaire	Core	Extended	Reference
**Demographics**	Age, gender, years in education, working status, weight, height	X	X	
**Characterisation of** **pain**	Presence and duration of pain	X	X	
**Family history**	Family history of chronic pain		X	
**Pain medication**	Currently taking pain medication	X	X	
	Brief Pain Inventory – Usefulness of medication		X	Cleeland and Ryan ^15^
	Adherence to medication		X	
**Pain severity**	Chronic Pain Grade	X	X	Von Korff, *et al*. ^16^
	Brief Pain Inventory – Pain Severity		X	Cleeland and Ryan ^15^
**Pain quality**	DN4 Questionnaire	X	X	Bouhassira, *et al*. ^9^
	DN4 Examination		X	
	Neuropathic Pain Symptom Inventory		X	Bouhassira, *et al*. ^17^
	PainDETECT		X	Freynhagen, *et al*. ^10^
**Pain location**	List of locations	X	X	
	Body map		X	
**Pain interference**	PROMIS Pain Interference		X	Cella, *et al*. ^19^
**Pain catastrophizing**	Pain Catastrophizing Scale	X	X	Sullivan, *et al*. ^20^
**Health status and** **quality of life**	EQ-5D-5L	X	X	Herdman, *et al*. ^21^
	PROMIS Depression	4a	8a	Cella, *et al*. ^19^
	PROMIS Anxiety	4a	8a	
	PROMIS Sleep Disturbance	4a	8a	
	PROMIS Fatigue		X	
	Trauma	X	X	
**Disease specific** **(diabetic neuropathy)**	Michigan Neuropathy Screening Instrument		X	Feldman, *et al*. ^18^
**Personality**	Ten Item Personality Inventory	X	X	Gosling, *et al*. ^22^
	International Personality Item Pool (Emotional Stability)		X	Goldberg ^23^
**Lifestyle**	Smoking	X	X	Campbell, *et al*. ^24^
	Alcohol	X	X	
	International Physical Activity Questionnaire		X	Craig, *et al*. ^25^

**Table 2.** Summary of tests performed during the DOLORisk protocol.

Cohort	Protocol	Neurological examination	TCSS	TNSn	Skin biopsy	QST	NCS	EEG	Threshold tracking	CPM
Population	Core									
Diabetes	Extended	X	X		X	X	X	X	X	X
Traumatic nerve injury	Extended	X	X				X		X	
Surgery	Extended	X				X			X	X
Chemotherapy	Extended	X		X		X	X		X	
Extreme phenotypes	Extended	X	X			X	X		X	

TCSS- Toronto clinical scoring system; TNSn- Total Neuropathy Score – Nurse; QST- Quantitative sensory testing; EEG - Electroencephalography; CPM- Conditioned pain modulation.

### Tools for phenotyping

**Questionnaires**

***Demographics***

Demographic information captured includes age, gender, weight, height, years in education, and working status.

***Characterisation of pain***

The presence and duration of pain (and also dysaesthesia) are assessed. Family history of chronic pain is recorded. Pain medication (individual drugs, e.g. paracetamol or gabapentin, and dosage), analgesic relief obtained and adherence to medication are recorded according to the Brief Pain Inventory (BPI) ^15^.

***Pain intensity***

Intensity of the pain is assessed with two questionnaires: the Chronic Pain Grade (CPG) ^16^ over the past three months, and the BPI’s subscale for assessment of average pain severity over 24 hours (which uses an 11 point numerical rating scale). One additional item asks about average pain over the past seven days.

***Pain quality***

Neuropathic descriptors of the pain are characterised with three tools: the DN4 ( *Douleur Neuropathique en 4 questions*) ^9^, the Neuropathic Pain Symptom Inventory (NPSI) ^17^, and the painDETECT ^10^. The Michigan Neuropathy Screening Instrument (MNSI) ^18^ is used specifically for diabetic neuropathy.

***Pain location***

The participants are asked to indicate in which body site they feel pain. This is assessed in two ways: a list of body sites and a body map. The participants are asked to identify all the body locations in which they experienced pain over the previous three months, and to mark the pain that bothers them the most. The body sites include: Back pain; Neck or shoulder pain; Facial or dental pain; Headache; Stomach ache or abdominal pain; Pain in the arms; Pain in the hands; Chest pain; Pain in the hips; Pain in the legs or knees; Pain in the feet; Pain throughout the body (widespread pain); Other pain. Using a list of body sites affected by pain provides alignment with pre-existing population cohorts, and is compatible with the recommendations of van Hecke, *et al*. ^1^. This will allow us to test the viability and feasibility of this approach to phenotyping neuropathic pain. Detailed body maps will be available for all participants in the extended protocol, which will provide additional accuracy and also enable direct comparison with the list of body sites.

The core and the extended protocols take a different approach to identify the location in which the participant should be asked to rate pain. The rationale for this is that the recommendation for grading neuropathic pain is based upon pain and clinical signs in a neuroanatomically plausible distribution ^26^. The core protocol is designed for the assessment of neuropathic pain of diverse aetiologies at population level, and there is no prior expectation as to the neuroanatomically plausible distribution. Then, participants are asked to specify body regions in which they experience pain, and choose one body region in which the pain bothers them most. In the core protocol, participants are asked to answer the questions that relates to pain intensity, quality and interference in respect to the body region in which pain bothers them most. The approach in the extended protocol is different because in these cohorts the likely aetiology of neuropathic pain is known and therefore the neuroanatomically plausible distribution is pre-determined. For instance in diabetic neuropathy or chemotherapy induced neuropathy the neuroanatomically plausible distribution is the feet, whereas following post-traumatic nerve injury the neuroanatomically plausible distribution is the innervation territory of the affected nerve. Participants are explicitly asked by the investigator to focus on the neuroanatomically plausible distribution when answering the questions on pain intensity, quality and interference. To capture information on other types of pain we then ask about pain in other body regions.

***Pain interference, quality of life and psychological variables***

The Patient-Reported Outcomes Measurement Information System (PROMIS) ^19^ questionnaires are used to assess various psychological and psychosocial variables. They include depression, anxiety, sleep disturbance, fatigue and pain interference. Two bespoke questions adapted from the existing population data ask about traumatic life experiences. The EQ-5D-5L ^21^ measures quality of life with a visual analogue scale and five items evaluating the impact of pain on the ability of the participant to perform everyday tasks.

Two questionnaires assessing personality and in particular neuroticism are included in the DOLORisk protocol. The Ten-Item Personality Inventory (TIPI) ^22^ evaluates extraversion, agreeableness, conscientiousness, neuroticism, and openness to experience. The 10-item International Personality Item Pool’s (IPIP) ^23^ representation of the Goldberg ^27^ markers for Emotional Stability offers a more precise characterisation of neuroticism. Pain catastrophizing behaviours are recorded through the Pain Catastrophizing Scale (PCS) ^20^.

***Lifestyle***

Smoking and alcohol are recorded according to Campbell, *et al.* ^24^ The short form of the International Physical Activity Questionnaire (IPAQ) ^25^ is included in the lifestyle variables to account for physical activity.

### Clinical assessment and specialised investigations

***Neurological examination***

A comprehensive structured upper and lower limb neurological examination is performed to detect clinical signs of a neurological lesion such as a peripheral neuropathy ^5, 28– 30^. The examination includes assessment of temperature (using Somedic RollTemp, Somedic AB, Sweden), light touch (using 10g monofilament) and pinprick sensation (using ‘Neurotip’), joint position sense (proprioception), vibration perception using a 128Hz tuning fork, deep-tendon reflexes (using a Queen square tendon hammer and recorded as present as normal, present with reinforcement, absent or brisk), muscle bulk, and motor power. The clinical findings for a length-dependent neuropathy are quantified with the Toronto Clinical Scoring System (TCSS) ^31^. The Total Neuropathy Score – Nurse (TNSn) ^32^ is used for chemotherapy-induced neuropathy. For other causes of neuropathic pain the spatial extent of sensory deficits and sensory hypersensitivity is recorded on a body map.

***Nerve conduction studies***

Nerve conduction tests, to confirm the presence of a length dependent neuropathy, are performed in line with those recommended by the American Academy of Neurology and American Association of Electrodiagnostic Medicine ^33, 34^. Sural sensory and peroneal motor nerve conduction studies are performed in one lower extremity. If both studies are normal ^33^ no further tests are performed. If either test is abnormal additional nerve conduction studies are performed that include: ipsilateral tibial motor nerve; contralateral sural sensory nerve, peroneal motor or tibial motor nerves; or ulnar sensory, median sensory, and ulnar motor nerves in one upper extremity. The minimum case definition criterion for electrodiagnostic confirmation of a length dependant neuropathy is an abnormality of any attribute of nerve conduction studies in two separate nerves, one of which is the sural nerve. Variables such as skin temperature, age, height, gender, and weight are measured and accounted for when interpreting nerve conduction tests. Nerve conduction tests are not repeated if study participants have previous results available which were performed within the last 2 years.

***Electroencephalography***

Electroencephalography (EEG) reflects the summated activity of synchronised arrays of brain neurons. Recent studies found correlations between several EEG parameters and pain perception in healthy subjects, suggesting that EEG can identify parameters that relate to the individual's way of processing pain. Examining EEG parameters in peripheral neuropathy patients can potentially identify those individual patterns of pain processing which are part of the array of factors that determine the final clinical pain phenotype ^35, 36^. Establishing EEG as an appropriate biomarker for pain perception relies on its accuracy to correctly classify subjects as belonging to the pain or no-pain conditions. In order to achieve this goal we follow the standard statistical steps of multivariate pattern analysis. A range of classifiers that distinguish the painful from the non-painful brain include measures of peak activity within the various EEG frequency bands per electrode, point to point connectivity between each of 64 electrodes, as well as identification of brain networks. This is expected to allow new understanding about the neurophysiological aspects of pain processing in the painful brain. The classification method finally employed will be the one with the highest classification accuracy on a test set after being trained on a separate training set.

***Threshold tracking***

Threshold tracking is an electrophysiological tool that assesses nerve excitability ^37^. Nerve excitability measures are determined by the biophysical properties of myelinated axons and the axon membrane potential. The information obtained about nerve properties is complementary to conventional nerve conduction studies: measurements of action potential amplitude and latency are limited indices of function, providing information only on the number of conducting fibres and the conduction velocity of the fastest, while threshold tracking is sensitive to membrane potential at the site of stimulation ^37^. In DOLORisk several measures of axonal excitability, such as refractoriness, supernormality, strength-duration time constant and threshold electrotonus, are assessed. The excitability measures are recorded from the motor and sensory divisions of the median nerve in line with published recommendations ^38^. This can also be used to model ion channel function. We will explore the relationship of these measures to the risk of developing neuropathic pain and the relationship to pain intensity. Training will be provided to clinicians performing threshold tracking measurements to ensure the reliability of the data and harmonisation of nerve excitability protocols in all centres.

***Skin biopsy for intra-epidermal nerve fibre assessment***

Intra-epidermal nerve fibre density (IENFD) is a validated tool for the assessment of small fibre pathology ^39^. In DOLORisk IENFD is determined from skin biopsy samples taken in accordance with published guidelines provided by the European Federation of Neurological Societies/Peripheral Nerve Society Guideline on the utilisation of skin biopsy samples in the diagnosis of peripheral neuropathies (see supplementary file: Skin biopsy for intraepidermal nerve fibre assessment) ^39^. The skin biopsies are taken at the end of the clinical assessment once all relevant investigations are completed. Participants do not under undergo a skin biopsy if they are on warfarin or found to have other contraindications.

***Quantitative sensory testing***

*Static tests*

Quantitative sensory testing (QST) is a measure of sensory perception in response to a defined sensory stimulus. This test can show abnormalities in sensory function and be used to generate a sensory profile in respect to different sensory modalities assessing both gain and loss of function. For bilateral neuropathic pain disorders such as peripheral neuropathy QST is performed unilaterally on the dorsum of the most affected foot. For unilateral neuropathic pain disorders QST is performed bilaterally in the affected area and the contralateral equivalent body region (which acts as a helpful comparator). QST is performed according to a modification of the previously published protocol of the German Research Network on Neuropathic Pain (DFNS) ^40^. These modifications were made in order to improve efficiency when performed in a restricted timescale. The wind up ratio (WUR) is not performed unless the patient is having conditioned pain modulation (CPM) tests in which case it will be helpful to have a measure of central sensitisation. WUR is performed on the forearm instead of the dorsum of the hand in order to minimise the influence of peripheral sensory loss on detection of central processes. Thermal sensory limen is performed in those patients with peripheral neuropathy but not in peripheral nerve injury (a situation where it is less informative). The assessment of mechanical pain sensitivity is shortened and two rounds of tests are performed instead of the five rounds included in the full DFNS protocol. All other tests are identical to the DFNS protocol and all study sites will be trained and certificated in the DFNS protocol to promote standardisation. QST data is entered into the data analysis system, Equista (version 1.2.2., CASQUAR GmbH), which was developed by the DFNS. Equista transforms the raw QST data into z-scores thus normalising for age, gender, and the body location of testing ^41, 42^. A z-score of zero is equal to the mean of the population. A score of greater or less than two standard deviations from the mean indicates gain of function or loss of function, respectively.

*Dynamic tests*

Conditioned pain modulation (CPM) provides insight into an individual’s endogenous analgesic mechanisms ^43, 44^. It can be assessed in a non-invasive manner and may be a key vulnerability factor for chronic pain and has also been shown to be predictive of treatment response. The protocol for CPM testing is in keeping with published recommendations (see supplementary file: Protocol for conditioned pain modulation) ^45, 46^.

***Genetics***

DNA is extracted from a whole blood sample collected at recruitment. The analysis will follow three complementary approaches: genome-wide association studies (GWAS); whole exome sequencing to identify rare, high-impact coding variants; and targeted sequencing of selected candidate genes.

### Definition of neuropathy

The participants who undergo the extended protocol are assessed for neuropathy (when this is considered a relevant possibility by the investigator e.g. a patient with diabetes) and in all cases are also graded for neuropathic pain. To diagnose peripheral neuropathy, we use the criteria outlined by Tesfaye, *et al.* ^47^ that classify neuropathy as possible, probable or confirmed:

Possible peripheral neuropathy is defined as the presence either of sensory symptoms, i.e. decreased sensation (e.g. “asleep, numbness”), positive neuropathic sensory symptoms (e.g. prickling or stabbing, burning or aching pain) predominantly in the toes, feet, or legs; or of sensory signs, i.e. symmetric decrease of distal sensation or unequivocally decreased or absent ankle reflexes.

Probable peripheral neuropathy corresponds to any two or more of the following: sensory symptoms (as above), decreased distal sensation, or unequivocally decreased or absent ankle reflexes.

Confirmed peripheral neuropathy is defined as the presence of an abnormality of nerve conduction studies and a sensory symptoms OR signs of neuropathy. If nerve conduction studies are normal, a validated measure of small fibre neuropathy (abnormal thermal thresholds on QST or reduced intra-epidermal nerve fibre density) may be used ^47^.

### Definition of neuropathic pain

The Neuropathic Pain Special Interest Group (NeuPSIG) of the International Association for the Study of Pain (IASP)’s grading for neuropathic pain ^48^ is used to grade neuropathic pain for all study participants recruited. Each study participant’s pain is assessed using these published criteria as below. Possible neuropathic pain must fulfil criteria 1 and 2. Probable neuropathic pain must fulfil criteria 1, 2 and 3. Definite neuropathic pain must fulfil all 4 criteria.

1. Pain with a distinct neuroanatomically plausible distribution, e.g. pain symmetrically distributed in the extremities – completion of body map and clinical history.
2. A history suggestive of a relevant lesion or disease affecting the peripheral or central somatosensory system – e.g. diagnosis of diabetes mellitus and a history of neuropathy symptoms including decreased sensation, positive sensory symptoms, e.g. burning, aching pain mainly in the toes, feet or legs.
3. Demonstration of distinct neuroanatomically plausible distribution of neuropathic pain – e.g. presence of clinical signs of peripheral neuropathy, i.e. decreased distal sensation or decreased/absent ankle reflexes.
4. Demonstration of the relevant lesion or disease by at least one confirmatory test – e.g. abnormality on either the nerve conduction tests or IENFD.

In the large, population-based cohorts, the core protocol permits the ‘entry level’ approximation to a classification of “possible neuropathic pain”, based on the NeuroPPIC phenotyping consensus ^14^. This includes positive responses to the DN4 screening questionnaire, and relevant site and severity of pain as outlined above. Additional information on diagnosis of any pain conditions will be available.

## Cohorts

**Figure 1.**
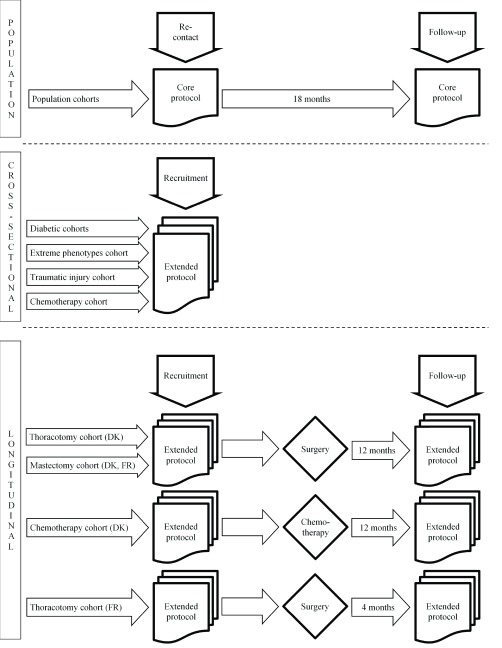
DOLORisk Recruitment flow. DK = Denmark, FR = France.

DOLORisk is a multi-centre cross-sectional and longitudinal observational study. Multiple cohorts with neuropathic pain from different causes will be included. Each cohort has its own specific inclusion and exclusion criteria, and follows a specific recruitment flow ( Figure 1; Table 3– Table 5).

**Table 3.** Inclusion and exclusion criteria for invitation to the population cohort for the DOLORisk protocol.

	Inclusion criteria	Exclusion criteria
**Population cohort**	• Previous participation with GoDARTS or GS:SFHS. • Existing consent to be re-contacted. • Identified as being currently alive. • Currently has a postal address on file. • ≥ 18 years.	• Unable to give consent. • No current postal address available. • Identified as having died.

**Table 4.** Inclusion and exclusion criteria for the cross-sectional cohorts for the DOLORisk protocol.

	Inclusion criteria	Exclusion criteria
**Peripheral** **neuropathic pain**	• ≥18 years with a diagnosis of peripheral neuropathy based on a prior clinical assessment combined with supportive clinical investigations such as abnormal nerve conduction studies, reduced intraepidermal nerve or abnormal findings on quantitative sensory testing. • Symptoms highly suggestive of neuropathy that in the judgement of the clinical researcher are suitable for the study even if they do not fulfil other inclusion criteria. • Patients who do not fulfil any of the exclusion criteria. • Diabetes cohorts: Type 1 or Type 2 diabetes	• Pregnant. • Incapacity to give consent or to complete the study questionnaires due to insufficient language command or mental deficiencies. • Concurrent severe psychological or psychiatric disorders. • Moderate to severe pain from other causes that may confound assessment or reporting of pain (e.g. spinal canal stenosis). • Central nervous lesions, which may complicate somatosensory testing. • Patients who are in the opinion of the investigator unsuitable for participation in the study.
**Extreme** **phenotypes**	• ≥16 years with a set of symptoms that resemble those seen on Paroxysmal Extreme Pain Disorder, Familial Episodic Pain Syndrome or Erythromelalgia. • Existing diagnosis of Paroxysmal Extreme Pain Disorder or Familial Episodic Pain Syndrome or Erythromelalgia. • Reduced pain sensibility. • First degree relatives of patients who meet diagnostic criteria for Paroxysmal Extreme Pain Disorder, Familial Episodic Pain Syndrome, Erythromelalgia or inability to experience pain. • Patients who do not fulfil any of the exclusion criteria.	• Pregnant. • Incapacity to give consent or to complete the study questionnaires due to insufficient language command or mental deficiencies. • Concurrent severe psychological or psychiatric disorders, especially severe claustrophobia. • Moderate to severe pain arising as a consequence of other disorders causing pain but that are not associated with those mentioned before as channelopathies. • Central nervous system diseased that may complicate the somatosensory testing. • Patients who are in the opinion of the investigator unsuitable for participation in the study. • Treatment or topical capsaicin cream/ ointment or Lidocaine patch within 30 days prior to Day 1 on the skin area that will be tested. • Presence of oedema or any skin condition at the ankle level that may interfere with the microneurography procedure.

**Table 5.** Inclusion and exclusion criteria for the longitudinal cohorts *for the DOLORisk protocol*.

	Inclusion criteria	Exclusion criteria
**Chemotherapy**	• ≥18 years. • Diagnosed with high-risk colorectal cancer. • Planned adjuvant treatment with oxaliplatin and flourouracil (5-FU) or capecitabine (Pro 5-FU).	• Known metastatic cancer. • Previous treatment with chemotherapy. • Significant mental illness. • Alcohol abuse. • Known diabetes. • Significant neuropathic diseases. • Spinal cord stenosis. • Peripheral vascular diseases (Fontaine >2). • Chronic pain with a pain intensity on a 0-10 numeric rating scale >5. • Patients who do not speak, read or understand Danish.
**Thoracotomy**	• ≥ 18 years. • Scheduled for lung cancer resection performed via thoracoscopy and/or thoracotomy, including lobectomy, bilobectomy, pneumonectomy, resection of the tracheobronchial bifurcation, wedge resection, sleeve resection and combinations hereof. • Willingness and ability to comply with study procedures as judged by the site investigator/ manager. • Expected availability for follow-up throughout the study.	• Mental incapacity or language barriers precluding adequate understanding of study procedures. • Current alcohol or substance abuse according to the site investigator’s medical judgement. • Unsuitability for participation in the study for any other reason, e.g. due to a significant serious underlying condition (e.g. other cancer or AIDS), as determined by the site investigator/manager. ADDITIONALLY in FR: • Previous surgery on the same area. • Surgery targeting only the pleura or mediastinum. • Peripheral neurological pathology or central (brain damage, multiple sclerosis) susceptible to interfere with the evaluation of the post-operative pain. • History of significant mental illness: psychosis, severe depression having motivated a hospitalisation, suicide attempt. • Current major depressive episode at the time of the evaluation. • Abuse of drug or psychoactive substance during the last six months. • Patients participating in another protocol of biomedical research.
**Mastectomy**	• Women ≥ 18 years. • Scheduled for breast cancer resection performed via lumpectomy (partial or segmental mastectomy) or mastectomy with or without sentinel lymph node biopsy and axillary lymph node dissection, and any combinations hereof. • Affiliated to a social security scheme. • Danish/French language (read, written and spoken). • Willingness and ability to comply with study procedures as judged by the site investigator. • Expected availability for follow-up throughout the study.	• Cognitive or psychological disorders incompatible with the respect and/or the understanding of the protocol. • Current alcohol or substance abuse according to the site investigator's medical judgement. • Unsuitability for participation in the study for any other reason, e.g. due to a significant serious underlying condition (e.g. other cancer or AIDS), as determined by the site investigator. • Previous surgery on the same area. • Peripheral neurological pathology or central (brain damage, multiple sclerosis) susceptible to interfere with the evaluation of the post-operative pain. • History of significant mental illness: psychosis, severe depression having motivated a hospitalisation, suicide attempt. • Current major depressive episode at the time of assessment. • Abuse of drug or psychoactive substance during the last six months • Participating in another protocol of biomedical research. • Other cancer or AIDS. • Scheduled for bilateral mastectomy. • Presence of chronic pain before the breast cancer surgery. • Workplace accident, litigation or search for compensation.

### Population cohort

Generation Scotland: the Scottish Family Health Study (GS:SFHS) ^49^ and Genetics of Diabetes Audit and Research Tayside (GoDARTS) ^50^ are population-based genetic epidemiology studies. DNA, socio-demographic and clinical data are available for 24,000 GS:SFHS participants and 20,000 (9,000 with diabetes) GoDARTS participants across Scotland. Participants will be contacted by post and invited to complete the DOLORisk core protocol. After 18 months, enrolled participants will be invited to complete the same questionnaire to assess development, progression or remission of any pain. For the population cohorts it is estimated that between 7% (GS:SFHS) and 25% (GoDARTS) of those with chronic pain will have neuropathic pain ^51^. Therefore, 1,500 participants with neuropathic pain and 3,000 controls are anticipated from GS:SFHS and 2,000 participants with neuropathic pain and 4,000 controls are anticipated from GoDARTS.

### Cross-sectional cohorts assessed with the extended protocol

Patients with peripheral neuropathic pain, e.g. diabetic neuropathy, chemotherapy-induced neuropathy, and traumatic nerve injury will be recruited by the University of Oxford, Imperial College London, Kiel University, Technion – Israel Institute of Technology, Neuroscience Technologies, and Aarhus University, from both primary and secondary care. Patients with extreme pain phenotypes, such as insensitivity to pain, will also be recruited. The study participants will be assessed as per the DOLORisk extended protocol.

### Longitudinal cohorts assessed with the extended protocol

Patients undergoing mastectomy, thoracotomy or receiving chemotherapy will be recruited by INSERM (French National Institute for Health and Medical Research) and Aarhus University. The surgical cohort of study participants will be recruited among patients scheduled for lung surgery or breast cancer surgery. The study participants receiving chemotherapy will be recruited from patients diagnosed with colorectal cancer. All study participants in this cohort will undergo the extended protocol before surgery or receiving chemotherapy. Thereafter, at different times ranging from 4 to 12 months participants will be re-assessed, using the extended protocol, to determine the development of neuropathic pain ( Figure 1). We expect to include 50 patients scheduled to undergo chemotherapy and 590 patients scheduled for lung or breast surgery.

## Data analysis

### Sample size calculation

The sample size for the protocol is largely based on the primary outcome, which is the number of participants to explore the genetic risk factors of neuropathic pain. The main comparison will be between those study participants diagnosed with neuropathic pain and those are diagnosed with no pain or pain of non-neuropathic nature. We will also be exploring physiological and psychosocial risk factors and these outcomes will require smaller sample sizes.

For example, based on the CaTS power calculator ^52^, we will have 80% power in an additive model with p=10 ^-8^, prevalence of neuropathic pain in the general population of 8%, with a disease allele frequency of 0.30 (GS:SFHS) or 0.38 (GoDARTS), and therefore a genotype relative risk of 1.34. Based on the CaTS GWAS power calculator ^52^, with 1,500 cases and 3,000 controls (as in the GS:SFHS cohort), we will have 82.7% power to identify SNP associations with a significance level of 5×10 ^−8^, assuming an additive model, a minor disease allele frequency of 0.3, a genotypic relative risk of 1.35, and a prevalence of the diabetic neuropathic pain in the general population of 10% ^1^.

For the extended phenotyping of painful versus painless diabetic neuropathy (estimating 1000 subjects in each group) we will have 80% power to detect an allelic odds ratio of 1.7 at genome wide significance level (p<5×10 ^-8^). We will also be able to cross-validate between these cohorts. We have identified a further cohort of diabetic neuropathy individuals in Sweden who will be available for replication genotyping. In collaboration with the SUMMIT consortium, we would also like to combine data across diverse diabetic complications in order to enhance the power to detect genetic determinants of the microvascular complications of diabetes.

Further sample size calculations have been performed depending on the individual outcome measures being measured.

***QST***

Sample size was determined according to the warm detection threshold data for patients with diabetes ^5^. This calculation revealed a minimum sample size of 34 was required per group for a power of >0.8 (difference in means 2.0; standard deviation 4.3; a = 0.05).

***CPM***

A cohort of 53 subjects gives an 80% power in between group differences of >0.25 standard deviations equivalent to 1.0 to 1.6 range on the 0-10 pain numerical rating scale using a typical QST parameter such as conditional pain modulation.

## Data management

The University of Dundee’s Health Informatics Centre (HIC) Services acts as a hub for data management. HIC Services develops bespoke software to support secure data collection, provides recruitment support for clinical studies and manages a data entry service. All services provided by HIC are delivered within a secure Safe Haven environment to ensure data are managed safely and in compliance with Data Protection legislation. All HIC processes are governed by approved Standard Operating Procedures. The questionnaire data is collected during the visit either on a paper clinical report form, or on a digital one, depending on the centres. The data is then entered by the investigator in the DOLORisk database through a bespoke interface. In order to limit input errors, the interface includes data checks and acceptable ranges, for instance for age, height and weight. Oxford have access to the whole dataset and perform checks on the quality and completeness of the entered data. Issues such as missing data are fed back to the respective centres so that they can be addressed.

GoDARTS and GS:SFHS datasets are already hosted on secure HIC servers. Participants’ identities will be shielded at all times from the research team, according to the secure SOPs.

External datasets generated by DOLORisk will be sent to HIC in anonymised format. When ready, these updated datasets will be transferred to the analytics platform held on a separate server and network from the HIC data management function within a remote-access Safe Haven for research projects. It has full analytical functionality including software (e.g. R and SAS) and is supported by powerful processing. Remote access to the Safe Haven analytics platform is available to approved project researchers, after they have signed appropriate agreements. No individual-level data can be removed from the Safe Haven, but summary outputs of analysis are released, after prompt screening by HIC to ensure that no potentially identifiable information is included to reduce the risk of accidental disclosure. Clinical phenotype data will be linked in anonymised format to genomic outputs.

## Ethics and dissemination

Ethic approvals were obtained at the national level. Details can be found in Table 6. Participants are included in the protocol only after having given their written informed consent. Their decision whether to take part, or withdrawal during the course of the study, in no way alters their normal medical care. The signed informed consent is obtained by the clinician in charge of the patient or the healthy volunteer.

**Table 6.** DOLORisk cohorts approvals. NT: Neuroscience Technologies. INSERM : Institut National de la Santé Et de la Recherche Médicale. CS: cross-sectional. Pro: prospective. REC: Research Ethics Committee. ANSM: Agence nationale de sécurité du médicament et des produits de santé (national agency for medicines and health products safety). CPP : Comité de protection des personnes (ethical research committee). CCTIRS: Comité consultatif sur le traitement de l'information en matière de recherche dans le domaine de la santé (advisory committee on data processing in health research). CNIL: Commission nationale de l’informatique et des libertés (data protection authority).

Centre	CS or Pro	Aetiology	Anticipated sample size	Ethics committee	Ethics reference	Registration link	Reference	End date
Dundee	Pro	Mixed	5500	Tayside Committee on Medical Research Ethics	05/S1401/89	https://www.hra.nhs.uk/planning- and-improving-research/ application-summaries/research- summaries/dolorisk-dundee/	Smith, *et al*. ^49^	April 2018
				Yorkshire & The Humber - South Yorkshire REC	15/YH/0285			
Dundee	Pro	Diabetes	3000	Tayside Committee on Medical Research Ethics	053/04	https://clinicaltrials.gov/ct2/show/ NCT02783469	Hebert, *et al*. ^50^	
				Yorkshire & The Humber - South Yorkshire REC	15/YH/0285			
Oxford	CS	Extreme phenotypes	100	NRES Committee London - Riverside	12/LO/0017	https://clinicaltrials.gov/ct2/show/ NCT02696746		January 2019
	CS	Diabetes	300	West London REC 3	10/H0707/35	https://clinicaltrials.gov/ct2/show/ NCT02672059	Themistocleous, *et al*. ^5^	June 2019
Imperial	CS	Diabetes	200	London - Bromley REC	16/LO/1470			
Kiel	CS	Mixed	200	Ethics Committee of the Faculty of Medicine of Kiel University	D454/16	https://clinicaltrials.gov/ct2/show/ NCT02666456		March 2019
Technion	CS	Diabetes	200	Helsinki Committee of Rambam Health Care Campus	0052-15-RNB	https://clinicaltrials.gov/ct2/show/ NCT02402361		July 2018
NT	CS	Diabetes	100	Clinical Research Ethics Committee (CREC) of idcsalud in Catalonia	2016/43-NEU- MC Mutual	https://clinicaltrials.gov/ct2/show/ NCT02985294		March 2019
	CS	Traumatic	100					
Aarhus	CS	Diabetes	350	Central Denmark Region Committees on Health Research Ethics	Diabetic neuropathy, 1- 10-72-130-16	https://clinicaltrials.gov/ct2/show/ NCT02947828		May 2018
	CS	Chemotherapy	70	Central Denmark Region Committees on Health Research Ethics	Chronic neuropathy following chemotherapy, 20110158	https://clinicaltrials.gov/ct2/show/ NCT02654691		April 2017
	Pro	Chemotherapy	50	Central Denmark Region Committees on Health Research Ethics	Acute and chronic neuropathy after oxaliplatin, 1-10- 72-154-16			
Aarhus	Pro	Post-surgical	250	Central Denmark Region Committees on Health Research Ethics	Understanding risk factors and determinants for neuropathic pain after lung or breast surgery, 1-10-72-254-16 and 1-10-72- 23-17	https://clinicaltrials.gov/ct2/show/ NCT03124511 https://clinicaltrials.gov/ct2/show/ NCT02960971		November 2019
INSERM	Pro	Post-surgical	340	ANSM	160106B-32, 160287B-32	https://clinicaltrials.gov/ct2/show/ NCT02944721		November 2020
				CPP	CPP/2-16, 16 03 18			
				CCTIRS	16-331bis, 16- 330bis			
				CNIL	2007306 v 0, 1251929 v 0			

Where possible, datasets will be made publicly available once the study is completed. Gene variants associated with neuropathic pain risk will be entered into the existing PainNetworks database ^53^ that undergoes longstanding curation by the London Pain Consortium. Transcriptional profiling data will be entered into painnetworks.org and ArrayExpress. We will enrich this with anonymised normative data on sensory profiling and physiological variables. It will be possible to download clinical screening tools from the DOLORisk website.

Findings will be communicated to the scientific community via peer-reviewed publications (open access), and presentations at conferences. DOLORisk has partnered with patient organisations supporting people with pain and neuropathy-related disorders such as Pain Association Scotland, the InDependent Diabetes Trust, and Fibromyalgia Action UK. The results of the study will be sent to the organisations periodically.

## Current study status

Recruitment started in 2016 and is ongoing in all centres. As of December 2017, 1,915 participants in GoDARTS and 7,240 participants in Generation Scotland have returned the questionnaires of the core protocol. 1,062 participants have been recruited throughout the rest of the centres according to the extended protocol. All recruitment and follow-up activities are expected to be completed by mid-2019.

## Data availability

No data are associated with this article.

## Supplementary material

**Protocol for conditioned pain modulation**

**Skin biopsy for intraepidermal nerve fibre assessment**
