# Supplementary material for: DOLORisk: study protocol for a multi-centre observational study to understand the risk factors and determinants of neuropathic pain
Source: Wellcome Open Res. 2019 Feb 1;3:63. Originally published 2018 May 29. [Version 2] doi: 10.12688/wellcomeopenres.14576.2 (PMC6364377; doi:10.12688/wellcomeopenres.14576.2)
Supplement: Supplementary file 1 [file wellcomeopenres-3-16455-s0000.tgz › 6e32290e-db8e-4580-9b9e-06a6f0d7db1e_Supp_material_1.docx]

Protocol for conditioned pain modulation

Instructions for test operator

A video version of this protocol can be found at the following URL: <https://www.youtube.com/watch?v=jL9GgdsyHtA>

Familiarization:

Apply pressure stimuli on the dominant trapezius. Apply one stimulus, and ask the subject to indicate pain onset.

Apply heat stimuli on the non-dominant volar forearm; baseline temperature is 32 ^o^C; stimulus increase rate is 2 ^o^C/sec. Give three trapezoid steps of 43, 45 and 47^o^C, staying 7 seconds at the peak, with 10 sec ISI (end to onset). Ask subject to rate pain magnitude.

Non-dominant hand will be briefly immersed to wrist level in cold water of 10^o^C. The subject should provide pain score. In case if the score was below 20 on 0-100 NPS, water temperature should be further decreased by steps of 2 ^o^C until it evokes pain score of at least 20. If rating is above 80, the water temperature should be increased in order to be within the range of 20-80 NPS.

Search for the pain 50:

Start with a triple stimulation of 45, 44 and 46 ^o^C, each for 7 sec, ISI of 10 sec. Move the probe slightly between stimuli, and get ratings. Determine pain50 temperature if data is sufficient for such interpolation, at a resolution of half a degree. If needed, add stimuli, with temperatures higher or lower than the 44-46 range, according to the responses received.

3 min break (at least).

CPM performance

'Standalone' test stimuli:

Place the heat probe on the dominant forearm (non-active). Give three pressure-algometer stimuli for pain threshold at the dominant trapezius muscle. Wait 3-5 seconds between stimuli. Then give one 20-sec long heat pain at pain50 level. Ask for pain ratings once plateau temp is reached (1^st^), and at 10^"^ (2^nd^) and 20" (3^rd^).

3 min break (at least, good time for questionnaires).

Conditioning + Test stimuli:

Immerse the non-dominant hand in water. After 10 seconds repeat the test stimulus, pressure and then heat, as above. Optional: At the end, get pain rating for the water pain beforehand is withdrawn.

CPM calculation:

For pressure – subtract averaged PPT scores: during the immersion minus stand alone.

For heat – conditioned averaged 2nd and 3rd heat scores minus standalone averaged 2nd and 3rd heat scores.
