# Supplementary material for: DOLORisk: study protocol for a multi-centre observational study to understand the risk factors and determinants of neuropathic pain
Source: Wellcome Open Res. 2019 Feb 1;3:63. Originally published 2018 May 29. [Version 2] doi: 10.12688/wellcomeopenres.14576.2 (PMC6364377; doi:10.12688/wellcomeopenres.14576.2)
Supplement: Supplementary file 2 [file wellcomeopenres-3-16455-s0001.tgz › 4ba29959-092a-43a3-800f-089889ba865e_Supp_material_2.docx]

Skin biopsy for intraepidermal nerve fibre assessment

The determination of intraepidermal nerve fibre density (IENFD) from skin biopsy samples is a validated and sensitive diagnostic tool for the assessment of small fibre neuropathies, including diabetic neuropathy^[[1]](#footnote-1)^. Punch biopsies of skin were performed immediately after the completion of QST. Skin biopsies were not taken from participants on warfarin or those who had other contraindications to skin biopsy. Biopsy samples were taken in accordance with the consensus document produced by the European Federation of Neurological Societies/Peripheral Nerve Society Guideline on the utilisation of skin biopsy samples in the diagnosis of peripheral neuropathies ^1^. Subcutaneous anaesthesia was achieved with lidocaine (1%, 1–1.2 ml) before the biopsy was taken under sterile conditions. Skin biopsies were taken 10 cm proximal to the lateral malleolus with a disposable 3mm punch biopsy circular blade (Stiefel Laboratories Inc, GSK Plc). The biopsy was fixed in fresh periodate-lysine-paraformaldehyde (2%) for 12-24 hours. Tissue was then washed in 0.1 M phosphate buffer and stored for 2–3 days in 15% sucrose in 0.1 M phosphate buffer. After embedding in O.C.T. (Fisher Scientific UK Ltd), the tissue was snap frozen and stored at −80°C.

Fifty-micrometre thin sections were cut on a cryostat and immunohistochemistry for PGP 9.5 was performed on free-floating sections using the immunoperoxidase method. Samples were randomly selected for staining done on 24 well plates allowing reagents complete penetration of floating samples. For the brightfield method, samples were washed with TBS and placed on an 5% Hydrogen Peroxide solution on ethanol, followed by blockade of non-specific protein binding with 4% Normal Donkey Serum, 0.5% Milk and 0.1% Triton X in TBS. Primary antibody rabbit anti-PGP (protein gene product) 9.5 Ab (1:15000; Ultraclone Ltd, Yarmouth, Isle of Wight, UK or 1:800 Zytomed Systems, Berlin, Germany) was incubated overnight at room temperature. After rinsing the samples with TBS secondary Biotinylated Goat Anti-Rabbit IgG Antibody (1:400; Vector Laboratories, Burlingame, CA, USA) was used followed by addition of VECTASTAIN ABC Kit (Vector Laboratories, Burlingame, CA, USA) for 1 hour. Samples were washed and transferred to DAB Peroxidase Substrate Kit, 3,3’-diaminobenzidine (Vector Laboratories, Burlingame, CA, USA) until a visible stain emerged. Samples were rinsed with distilled water and progressively dehydrated with ethanol. Samples were mounted using DPX mounting media. PGP 9.5-immunoreactive nerve fibres crossing the basal membrane of the epidermis were counted under a 40x objective and a measurement of the length of the sample was also obtained. IENFD was assessed using a double bright-field microscope at 40X magnification using established counting rules and expressed as fibres per millimetre of epidermal length. IENFD were considered abnormal if below the fifth centile for age and gender matched healthy controls^[[2]](#footnote-2)^.

1. Lauria, G., S. T. Hsieh, O. Johansson, W. R. Kennedy, et al.: European Federation of Neurological Societies/Peripheral Nerve Society Guideline on the use of skin biopsy in the diagnosis of small fiber neuropathy. Report of a joint task force of the European Federation of Neurological Societies and the Peripheral Nerve Society. *Eur J Neurol* 2010:17(7): 79-92 10.1111/j.1468-1331.2010.03023.x [↑](#footnote-ref-1)
2. Lauria G1, Bakkers M, Schmitz C et al.: Intraepidermal nerve fiber density at the distal leg: a worldwide normative reference study. *J Peripher Nerv Syst*. 2010: 15(3):202-7 10.1111/j.1529-8027.2010.00271.x. [↑](#footnote-ref-2)
