# Supplementary material for: DOLORisk: study protocol for a multi-centre observational study to understand the risk factors and determinants of neuropathic pain
Source: Wellcome Open Res. 2019 Feb 1;3:63. Originally published 2018 May 29. [Version 2] doi: 10.12688/wellcomeopenres.14576.2 (PMC6364377; doi:10.12688/wellcomeopenres.14576.2)
Supplement: Peer review history

# Peer review: Referee response for version 2

**DOI:** 10.21956/wellcomeopenres.16455.r34754

**Published:** 2019-02-04

**Authors:** Simon Haroutounian

**Affiliations:** 1 Department of Anesthesiology and Washington University Pain Center, Washington University School of Medicine, St. Louis, MO, USA

The authors have addressed all my previous comments.

I have read this submission. I believe that I have an appropriate level of expertise to confirm that it is of an acceptable scientific standard.

# Peer review: Referee response for version 1

**DOI:** 10.21956/wellcomeopenres.15867.r33852

**Published:** 2018-11-05

**Authors:** Miroslav Backonja

**Affiliations:** 1 University of Washington, Seattle, WA, USA

This is a multicentre, international, prospective observational study with the goal to investigate in a systematic manner neuropathic pain due to multiple aetiologies targeting to enrol large number of patients, especially those with diabetic neuropathy.  Authors state that the primary objective is to identify a number of factors that would influence and lead to development of neuropathic pain and the association of genetic factors with risk of developing and maintaining neuropathic pain.

 This is a very ambitious and large undertaking, likely to generate a large amount of information and hopefully be a great source of publications.

 A lot of thought and work has been put into this project and a few things would be worth attending to in attempt to make it more productive. These would be comments in addition to those expressed by Dr. Haroutounian with which I agree.

 Statement of objectives is very general and imprecise, such as inclusion of environmental factors and none is ever addressed in the grant. It is not clear if this is matter of language used or lack of focus but for the reader of this project more precise definition of Objectives would be more meaningful.

 Regarding the location of pain, the current description is detailed but probably not meaningfully useful and it is unlikely to lead to interpretable results. Yes, it will be possible to check of boxes as to location of pain but the current schemata will not provide any useful information. 

 Foundation for conducting studies with EEG and threshold tracking is lacking and though rationale is presented it is scientifically insufficient at best and very weak.

I have read this submission. I believe that I have an appropriate level of expertise to confirm that it is of an acceptable scientific standard.

# Response

**Published:** 2019-01-28

**Authors:** Mathilde Pascal

**Affiliations:** Nuffield Department of Clinical Neurosciences, University of Oxford, UK

Thank you for your feedback. We have amended the article based on Dr Haroutounian’s comments, which you said you agreed with – please see our response to him in addition to our reply to you below.

*1. Statement of objectives is very general and imprecise, such as inclusion of environmental factors and none is ever addressed in the grant. It is not clear if this is matter of language used or lack of focus but for the reader of this project more precise definition of Objectives would be more meaningful.*

Thank you for this point. We intended “environmental” to mean “non-genetic”.  We agree that in this sense, it is indeed not used correctly when used for the first time in the description of the objectives. We believe the other uses are appropriate. We apologise for the confusion of the first use of the word, which we have now deleted.

*2. Regarding the location of pain, the current description is detailed but probably not meaningfully useful and it is unlikely to lead to interpretable results. Yes, it will be possible to check of boxes as to location of pain but the current schemata will not provide any useful information.*

The core protocol has a list of locations but the extended protocol uses this list and additionally a body map for the patient to complete, which will provide detailed information. The list of locations is a pragmatic approach in large questionnaire based surveys and was used to allow harmonisation with Generation Scotland and other ongoing epidemiological studies. This approach is consistent with the recommendations of the Neuropathic pain phenotyping by consensus recommendations of NeuPSIG  (van Hecke et al, 2015). As part of our analysis, we are testing this approach to phenotype neuropathic pain, and intend to publish an analysis of the validity and feasibility of the approach. We have the advantage that in the extended cohort we can directly assess the outcome of using a list of locations versus the use of a body map. We will revise any future phenotyping efforts accordingly.

*3. Foundation for conducting studies with EEG and threshold tracking is lacking and though rationale is presented it is scientifically insufficient at best and very weak.*

We have now expanded on both these points in the protocol in order to provide a clearer rationale for these investigations

# Peer review: Referee response for version 1

**DOI:** 10.21956/wellcomeopenres.15867.r33285

**Published:** 2018-06-15

**Authors:** Simon Haroutounian

**Affiliations:** 1 Department of Anesthesiology and Washington University Pain Center, Washington University School of Medicine, St. Louis, MO, USA

Pascal and colleagues present a protocol for a multicenter observational study (DOLORisk) for understanding risk factors and determinants of neuropathic pain of various etiologies. The authors should be congratulated for this massive and ambitious undertaking - the study is likely to provide enormous insight into risk factors associated with the development of neuropathic pain.

 Below are several comments authors may want to consider:

 The first point is related to my response related to "partial" presentation of sufficinet details of methods in order to be replicated.

 Some of the multiple assessment in the protocol, even when following guidelines, can have certain methodological variability.

 Procedures such as CPM and skin biopsy refer to general guidelines,  and may be challenging to reproduce without more detailed instructions. It would be advisable if the authors added an appendix with a summary of the specific CPM protocol, and skin biopsy protocol.

Additional comments:

 "Characterization of pain" section: It is unclear whether the medications will be recorded as individual drugs, as therapeutic groups vs pharmacological classes, and whether doses/duration will be recorded. It would be useful if the researchers provide the details in the protocol, and to describe how they intend to present these data.

 In "nerve conduction studies" section the last sentence notes that if prior nerve conduction tests are available, repeat tests will not be performed. Do authors intend to assign an acceptable window to the timeline? i.e. - if a patient has had DPN for 10 years, and has NCV results from 8 years ago, would those considered valid for the study purposes?

 Data management: it is not clear if there is a computer-based data collection system with direct data entry from each site, which then undergoes QC, or whether the data are collected on paper-pencil CRFs, then entered to a some electronic format (e.g. spreadsheets) and transmitted (via email?) to the University of Dundee HIC Services. This is important for controlling the steps where potential errors can occur, and it would be useful to provide more specific steps of data collection, entry and central storage.

 Table 5: the exclusion criteria for thoracotomy and mastectomy cohorts are quite different in terms of components not related directly to the surgery (e.g. cognitive disorders, substance use). Is there a rationale for these differences?

 Table 5: the sentence "Receiving another treatment than oxaliplatin and flourouracil (5-FU) or capecitabine (Pro 5-FU)." might be better worded as "receiving treatment other than oxaliplatin...". In addition, the adjuvant FOLFOX regimen often includes Leucovorin. For such "hard" criteria of excluding anything besides 5-FU/Capecitabline and oxaliplatin, the investigators may want to add leucovorin. Also - some centers add bevacizumab to adjuvant FOLFOX regimens - consider either allowing or explicitly excluding these patients.

I have read this submission. I believe that I have an appropriate level of expertise to confirm that it is of an acceptable scientific standard, however I have significant reservations, as outlined above.

# Response

**Published:** 2019-01-28

**Authors:** Mathilde Pascal

**Affiliations:** Nuffield Department of Clinical Neurosciences, University of Oxford, UK

Thank you for taking the time to read our study protocol and for your constructive feedback. We have addressed your comments below.

*1. The first point is related to my response related to "partial" presentation of sufficinet details of methods in order to be replicated. Some of the multiple assessment in the protocol, even when following guidelines, can have certain methodological variability. Procedures such as CPM and skin biopsy refer to general guidelines,  and may be challenging to reproduce without more detailed instructions. It would be advisable if the authors added an appendix with a summary of the specific CPM protocol, and skin biopsy protocol.*

The harmonisation of the protocols across our centres is absolutely essential in DOLORisk and we appreciate your feedback to make this protocol more useful and reproducible to other people. We have added the CPM protocol and the skin biopsy protocol in the appendices of the article. The CPM protocol also exists in video format: https://www.youtube.com/watch?v=jL9GgdsyHtA.

*2. "Characterization of pain" section: It is unclear whether the medications will be recorded as individual drugs, as therapeutic groups vs pharmacological classes, and whether doses/duration will be recorded. It would be useful if the researchers provide the details in the protocol, and to describe how they intend to present these data.*

Yes we are recording individual drugs. Wherever possible the dosage is also indicated. This section has been amended to further clarify this point.

*3. In "nerve conduction studies" section the last sentence notes that if prior nerve conduction tests are available, repeat tests will not be performed. Do authors intend to assign an acceptable window to the timeline? i.e. - if a patient has had DPN for 10 years, and has NCV results from 8 years ago, would those considered valid for the study purposes?*

Indeed, we take into account the results of previous nerve conduction studies if they are less than two years old, regardless of how long a participant has had DPN for. We have now added this clarification to the article.

*4. Data management: it is not clear if there is a computer-based data collection system with direct data entry from each site, which then undergoes QC, or whether the data are collected on paper-pencil CRFs, then entered to a some electronic format (e.g. spreadsheets) and transmitted (via email?) to the University of Dundee HIC Services. This is important for controlling the steps where potential errors can occur, and it would be useful to provide more specific steps of data collection, entry and central storage.*

The questionnaire data are collected during the visit either on a paper clinical report form, or on a digital one, depending on the participating centre. The data are then entered manually by the investigator in the DOLORisk database through a bespoke interface. In order to limit input errors, the interface includes data checks and acceptable ranges, for instance for age, height and weight. Oxford have access to the whole dataset and perform checks on the quality and completeness of the entered data. Issues such as missing data are sent back to the respective centres so that they can be addressed. The section on data management has been amended to include this information.

*5. Table 5: the exclusion criteria for thoracotomy and mastectomy cohorts are quite different in terms of components not related directly to the surgery (e.g. cognitive disorders, substance use). Is there a rationale for these differences?*

Most criteria are common between the mastectomy cohort and the thoracotomy cohort. Only point 4 (history of significant mental illness) might make a real difference between the French and the Danish cohort (some criteria are specific to procedure such as surgery targeting the pleura in thoracotomy). The reason for adding the exclusion criteria of significant mental illness is that our French centre wanted to avoid having problematic patients in terms of psychiatric morbidities. However the Danish centre is recording co-morbidities and we can exclude these cases at the analysis stage to match the French cohort if we feel that this is required for alignment.

The exclusion criterion of “cognitive or psychological disorders incompatible with the respect and/or the understanding of the protocol” in the mastectomy cohort is mirrored in the thoracotomy cohort by “mental incapacity or language barriers precluding adequate understanding of study procedures.” To clarify how the criteria match between the two cohorts, we have re-arranged the order of the exclusion criteria in the mastectomy cohort.

*6. Table 5: the sentence "Receiving another treatment than oxaliplatin and flourouracil (5-FU) or capecitabine (Pro 5-FU)." might be better worded as "receiving treatment other than oxaliplatin...". In addition, the adjuvant FOLFOX regimen often includes Leucovorin. For such "hard" criteria of excluding anything besides 5-FU/Capecitabline and oxaliplatin, the investigators may want to add leucovorin. Also - some centers add bevacizumab to adjuvant FOLFOX regimens - consider either allowing or explicitly excluding these patients.*

We do allow leucovorin (although rare at the centre where the standard is oxaliplatin iv and capecitabine as tablet (XELOX)) while bevacizumab is not used as adjuvant therapy in Denmark, which is the centre recruiting the chemotherapy cohort. However, what we actually meant with the criteria was in fact “Not receiving oxaliplatin and flourouracil (5-FU) or capecitabine (Pro 5-FU)”, but as this is already an inclusion criteria, we have now deleted this sentence.
